# The impact of dichotomization on network recovery

**DOI:** 10.3758/s13428-025-02861-6

**Published:** 2025-11-11

**Authors:** Nikola Sekulovski, Tessa F. Blanken, Jonas M. B. Haslbeck, Maarten Marsman

**Affiliations:** Department of Psychology, University of Amsterdam, Nieuwe Achtergracht 129B, PO Box 15906, 1001 NK Amsterdam, The Netherlands

**Keywords:** Bayesian graphical modeling, Dichotomization, Network psychometrics, Ordinal variables

## Abstract

Graphical models have become an important method for studying the network structure of multivariate psychological data. Accurate recovery of the underlying network structure is paramount and requires that the models are appropriate for the data at hand. Traditionally, Gaussian graphical models for continuous data and Ising models for binary data have dominated the literature. However, psychological research often relies on ordinal data from Likert scale items, creating a model-data mismatch. This paper examines the effect of dichotomizing ordinal variables on network recovery, as opposed to analyzing the data at its original level of measurement, using a Bayesian analysis of the ordinal Markov random field model. This model is implemented in the R package bgms. Our analysis shows that dichotomization results in a loss of information, which affects the accuracy of network recovery. This is particularly true when considering the interplay between the dichotomization cutoffs used and the distribution of the ordinal categories. In addition, we demonstrate a difference in accuracy when using dichotomized data, depending on whether edges are included or excluded in the true network, which highlights the effectiveness of the ordinal model in recovering conditional independence relationships. These findings underscore the importance of using models that deal directly with ordinal data to ensure more reliable and valid inferred network structures in psychological research.

## Introduction

Network psychometrics is a methodology that uses Markov random field graphical models (MRF, Kindermann & Snell, 1980) to examine the network structure in multivariate psychological data (Borsboom et al., 2021; Marsman & Rhemtulla, 2022; Robinaugh, Hoekstra, Toner, & Borsboom, 2020). In these models, nodes represent psychological variables, and included edges between pairs of nodes indicate conditional dependency relationships (Kindermann & Snell, 1980). Consequently, identifying the true network structure – the specific configuration of included and excluded edges – is a crucial aspect of analyzing these models.

To correctly recover the network structure, it is important that the estimated models are appropriate for the data at hand. So far, the most popular MRFs used in network psychometrics have been the Gaussian graphical model (GGM; Lauritzen, 2004) for continuous data, the Ising model (Ising, 1925; van Borkulo et al., 2014) for binary data, and the mixed graphical model for mixed continuous, categorical, and count variables (Haslbeck & Waldorp, 2015). However, while the GGM assumes that the data are continuous, most psychological variables are measured at the ordinal level due to the ubiquitous use of Likert-scale items. Copula Gaussian graphical models for binary, ordinal, and continuous data (CGGMs; Dobra & Lenkoski, 2011), have been proposed as one solution, although they have been more difficult to interpret because, unlike MRFs, CGGMs define conditional independence relations on latent continuous variables and specify a full joint distribution via a copula function. In this approach, a zero in the estimated precision matrix does not guarantee that the measured ordinal variables are conditionally independent (Dobra & Lenkoski, 2011; Liu, Lafferty, & Wasserman, 2009). Another practical solution is to use polychoric or Spearman correlations in GGMs without defining a likelihood on the ordinal scale. As with the CGGM, a zero in the estimated precision matrix does not guarantee that the measured ordinal variables are conditionally independent, and may lead to biased or unstable estimates, especially in small samples (Isvoranu & Epskamp, 2023).

The broad availability of user-friendly software for analyzing (C)GGMs (e.g., Mohammadi & Wit, 2019; Williams & Mulder, 2020), the Ising model (e.g., Marsman, Huth, Waldorp, & Ntzoufras, 2022; van Borkulo, Epskamp, & Robitzsch, 2016), as well as GGMs based on non-Pearson correlations (Epskamp, Borsboom, & Fried, 2018), has led to their almost exclusive use in network psychometrics.^1^ As a result, there has been a mismatch between the models commonly used in network psychometrics and the ordinal data typically encountered in psychological research. To address this mismatch, researchers are left with a number of workarounds: (i) treating ordinal data as continuous and applying the GGM; (ii) assuming latent Gaussianity or plugging in polychoric or Spearman correlations into the GGM; or (iii) dichotomizing the ordinal variables and using the Ising model. Despite broad agreement that dichotomizing variables is rarely justifiable (e.g., MacCallum, Zhang, Preacher, & Rucker, 2002), some researchers, faced with a lack of suitable models, might reasonably opt to dichotomize their ordinal (or skewed) variables rather than buy into continuity assumptions on the latent space (by using the CGGM) or use non-Gaussian correlations in the GGM. For example, Mullarkey, Marchetti, and Beevers (2019) dichotomized skewed ordinal data on adolescent depression, and Boschloo, Schoevers, van Borkulo, Borsboom and Oldehinkel (2016) dichotomized sum scores of emotional and behavioral problems in preadolescents due to violations of normality and linearity, which prevented the use of a GGM. Likewise, Cheung et al. , (2021) and Cai et al. , (2022) dichotomized depressive symptom items due to the lack of models for trichotomous data, and Zavlis et al. , (2022) used clinical cutoffs to dichotomize symptom scores for depression, anxiety, and traumatic stress. Garcia-Mondragon et al. , (2022) dichotomized cumulative trauma scores at the 75th percentile. In all these cases, the Ising model was used to estimate network structures, reflecting the practice of dichotomizing ordinal variables in the absence of appropriate alternatives.

This situation changed when Marsman, van den Bergh, & Haslbeck (2025) proposed a MRF that models variables directly on the ordinal scale, without requiring data transformation. The authors introduced a fully Bayesian approach based on Bayesian model averaging (BMA; Hinne, Gronau, van den Bergh, & Wagenmakers, 2020; Hoeting, Madigan, Raftery, & Volinsky, 1999) to estimate both the structure and parameters of the model. This eliminates the need for ad hoc dichotomization and provides a statistically appropriate graphical model for ordinal data. The ordinal MRF approach is particularly attractive because it (a) yields a fully specified model for the joint distribution of ordinal variables, incorporating both interaction and marginal parameters, (b) avoids reliance on latent Gaussian assumptions or transformations, and (c) offers a fully Bayesian framework that quantifies uncertainty in both the network structure and the model parameters (e.g., Huth et al., 2023; Marsman et al., 2022; Sekulovski et al., 2024).

Thus, while the ordinal MRF can directly model the data without transformation, ensuring higher statistical efficiency, dichotomizing the data could potentially compromise the robustness of the recovered network structure, leading to a smaller precision when having the same sample size. Here, we explore such statistical consequences of dichotomizing ordinal data for network structure recovery. Therefore, the purpose of this paper is to illustrate the extent to which researchers would benefit in recovering the true network structure, from using the ordinal MRF to model their data as opposed to dichotomizing the ordinal variables and using the Ising model.

To evaluate the potential negative impact of dichotomization on network recovery, we simulate data from the ordinal MRF model, apply various dichotomization criteria to the variables, and compare how well the true network structure is recovered using models fitted to the dichotomized data versus those fitted to the original ordinal data. We focus exclusively on the ordinal MRF and its associated Bayesian analysis, as it (i) defines a proper Markov random field for ordinal and binary variables, and (ii) accounts for the uncertainty in both the network structure and its parameters. We do not consider approaches based on polychoric or Spearman correlations, as these do not yield a proper MRF for the observed ordinal data. We also do not include the methods implemented in the R packages BGGM (Williams & Mulder, 2020) or BDgraph (Mohammadi & Wit, 2019), which provide a Bayesian analysis of graphical models used in network psychometrics. Their implementations rely on the copula Gaussian graphical model (CGGM) to handle non-Gaussian variables. Furthermore, the Bayesian approach implemented in BGGM infers the network structure by testing individual edges, without accounting for the uncertainty in the remaining network structure (for a detailed discussion, see Sekulovski et al., 2024).

The next section provides a brief overview of the ordinal MRF and its Bayesian analysis. We then report the results of the simulation study and conclude with a discussion of our findings.

## The Bayesian analysis of the ordinal Markov random field

The ordinal Markov random field (Marsman et al., 2025) is a graphical model that describes the joint distribution of ordinal variables with potentially varying numbers of categories. The model parameters are edge weights, representing the strength of the partial associations between pairs of variables for which an edge is included, and category threshold parameters, accounting for the remaining variability that cannot be explained by the other variables in the network. For further details and references on this model, we refer the interested reader to Appendix A.

When analyzing psychometric network models, there are two sources of uncertainty: (i) the standard parameter uncertainty for the edge weight parameters, and (ii) the uncertainty in the network structure (see e.g., Huth et al., 2023; Sekulovski et al., 2024). Bayesian variable (edge) selection (e.g., Marsman et al., 2022; 2025; McCullagh, 1994; O‘Hara and Sillanpää, 2009) addresses the uncertainty in the network structure by stipulating prior distributions over both the network structure and the edge parameters. The resulting joint posterior distribution expresses the probability of the parameters and structures after seeing the data. In this paper, we focus on the posterior inclusion probability of each edge, which quantifies the evidence for edge inclusion after seeing the data. The posterior inclusion probability is subsequently used to compute the inclusion Bayes factor, quantifying the strength of evidence favoring either the inclusion or exclusion of edges (Sekulovski et al., 2024).

This approach, which we refer to as Bayesian graphical modeling, enables researchers to hierarchically account for uncertainty in both the network structure and the associated parameters. For example, when evaluating whether an edge is included between two variables in a three-variable network, one must consider all possible configurations of the remaining relations, resulting in a total of eight possible network structures (c.f., Huth et al., 2023; Sekulovski et al., 2024). In such cases, assessing the presence of a specific edge requires conditioning on the uncertainty of the rest of the network structure, followed by estimating the corresponding edge weight parameter. The Bayesian graphical modeling framework provides a coherent and principled way to achieve this. Software implementations are available in the R package bgms (Marsman, Arena, Huth, Sekulovski & van den Bergh, 2023), as well as the user-friendly wrapper package easybgm (Marsman, Huth, Keetelaar, Sekulovski, & van den Bergh, 2025). Further details and references are provided in Appendix A.

## Simulation study

To evaluate how much information researchers gain by using the ordinal MRF as opposed to dichotomizing their data and fitting the Ising model, we perform a small simulation study. For all analyses, we use the R package bgms.

### Design and analysis

We generate ordinal data for $p = 15$ variables, each with 5 ordinal categories coded from 0 to 4. Within the simulation design, we vary the number of observations *n* from 100 to 2, 000 in increments of 100. To examine how the marginal distribution of the ordinal categories affects model performance when the data are dichotomized, we simulate three distinct types of distributions across the $p = 15$ variables: (1) a strongly skewed distribution, where most responses fall in the lowest categories (henceforth referred to as *large skew*); (2) a mildly skewed distribution, where responses are more frequent in the lower categories (referred to as *mild skew*); and (3) a uniform distribution, where the frequencies of the ordinal categories are approximately equal (referred to simply as *uniform*).^2^ Skewness is common in measures of negative affect or symptom severity in healthy populations, making it an important condition to investigate.

In all simulation settings, we keep the same underlying network structure, with an overall network density of around 10%, with all the included edges having an edge weight parameter of 0.25. We simulate 100 data sets for each simulation condition. We deliberately use a fixed sparse network structure, as this allowed us to generate diverse ordinal category distributions while still maintaining sufficiently large edge-weight parameters for the included edges in the network. In Appendix B we provide the details of the data-generating process.

That the particular choice of the threshold for dichotomizing the data has an impact on parameter and structure recovery has been empirically demonstrated by Hoffman, Steinley, Trull, and Sher (2018). The authors illustrate how the threshold used to dichotomize the data and classify a symptom as active has an impact on the estimated parameters and network structure. Here we study this question more systematically, by dichotomizing the ordinal data using four different splits:

A 0 vs. 1:4
B 0:1 vs. 2:4
C 0:2 vs. 3:4
D 0:3 vs. 4.

We estimate the MRFs for the original ordinal data using the ordinal MRF and for the dichotomized data using the Ising model from all four splits, employing the R package bgms with the default prior settings (Appendix A).

Each model was analyzed using 10, 000 MCMC iterations with the first 1, 000 iterations as burn-in. For the dichotomized data sets in the condition with a large skew, four and 65 models failed during the estimation process for dichotomization criteria C and D, respectively. This is due to the fact that, under these extreme splits, the low frequency of higher categories often results in variables with only a single observed value (typically 0). In such cases, parameters are not identifiable due to the lack of variation in the data. Most of the affected data sets occurred under the sample size condition of $n = 100$, with a few additional cases in the $n = 200$, $n = 300$, and $n = 400$ conditions.

Before obtaining the posterior edge probabilities, we average over the 100 replications to approximate performance outcomes in the population, ensuring that the results are not driven by sampling variation. For the failed data sets described above, we replaced the failed models with successfully estimated models from within the same simulation condition. As a performance measure, we compute the Brier score (Brier, 1950), which quantifies the mean squared difference between the estimated posterior inclusion probabilities and the actual outcomes, with lower scores indicating better performance in recovering the true network structure. A Brier score of 0 indicates perfect accuracy, where the estimated posterior edge probabilities perfectly match the true inclusion, while a Brier score of 1 indicates the worst possible accuracy. Let $\pi$ be the estimated inclusion probability for the edge between two variables, and *t* be the true inclusion (either 0 if the edge is excluded or 1 if the edge is included), then the average Brier score for *N* edges is defined as:

$$ \text {BS} = \frac{1}{N} \sum _{i=1}^{N} \left( \pi _{i} - t_{i} \right) ^2. $$

To summarize, in the simulation, we vary the sample size, the marginal distributions of the ordinal variables, and the cutoff points used to dichotomize the data. We then compute Brier scores for the results in each of these simulation conditions. The code to reproduce the results is publicly available in an OSF repository at https://osf.io/9bzet/.

### Results

**Fig. 1:**
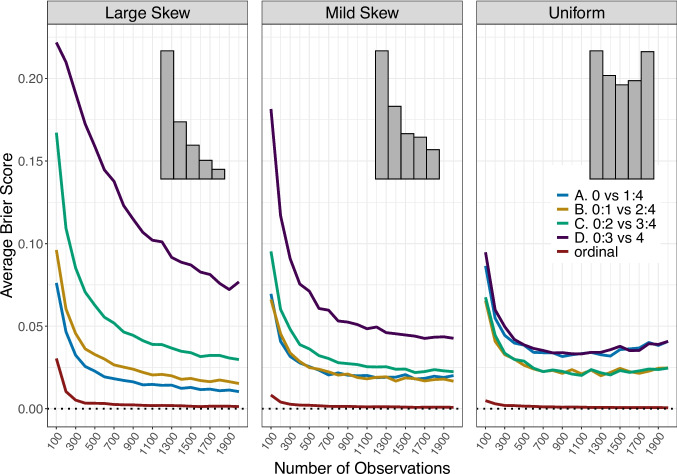
Brier scores for the accuracy of the models estimated using the ordinal data and the dichotomized data with the four different splits (indicated by different colors) are plotted as a function of the number of observations. The three panels represent the different distributions of the ordinal categories in the simulated data. Within each panel, an example histogram illustrates the distribution of the ordinal variables. These histograms were generated by randomly sampling one variable from each skewness condition and plotting its observed distribution. The *dotted black line* indicates a Brier score of zero, representing perfect recovery of the true network structure

Figure 1 shows the average Brier scores calculated for models estimated using ordinal data and four dichotomization cutoffs, plotted as a function of the number of observations. Across the three types of marginal distributions (displayed in the three panels), the model estimated on the ordinal data consistently achieves the best recovery of the network structure.

Analyzing the results separately for each marginal distribution of the ordinal variables, we see that when the ordinal variables have large skew (left panel of Fig. 1), there is a substantial inaccuracy in recovering the network structure based on the dichotomized data, especially for the more extreme splits. More specifically, and as expected, split D (purple) performs the worst, as it dichotomizes already skewed data in a way that drastically reduces variance, thereby impairing the model’s ability to accurately recover the network structure. Performance improves slightly for the remaining splits – C (green), B (yellow), and A (blue), respectively. Finally, we can see that when using the ordinal data directly (red), the ordinal MRF performs best in recovering the network structure; however, it still shows some inaccuracy for very small sample sizes. This is expected given the strong skew in the ordinal categories.

Next, when the ordinal categories exhibit a milder skew relative to the previous setting (middle panel of Fig. 1), we observe a very similar pattern. The overall inaccuracy is consistently lower compared to the large skew condition. The ordering of the dichotomization splits remains the same, and again, the model based on the ordinal data performs best. Notably, its performance is more stable and accurate even at lower sample sizes.

Finally, when the ordinal categories are roughly uniformly distributed (right panel of Fig. 1), all models perform better relative to their counterparts estimated on skewed data. The ordering of the different dichotomization splits remains the same, and now we observe that the models estimated on the ordinal data recover the true network structure almost perfectly, even for small sample sizes.

In all three panels, we observe that as the sample size increases, the models based on ordinal data (relatively quickly) reach an average Brier score of approximately 0, meaning they perfectly recover the true network structure. However, for the dichotomized data, we see that while accuracy initially improves with increasing sample size, it eventually levels off and remains constant, at least within the range of sample sizes explored in this simulation. To further investigate this pattern, we next explore the results by splitting them by the true inclusion of the edges.

**Fig. 2:**
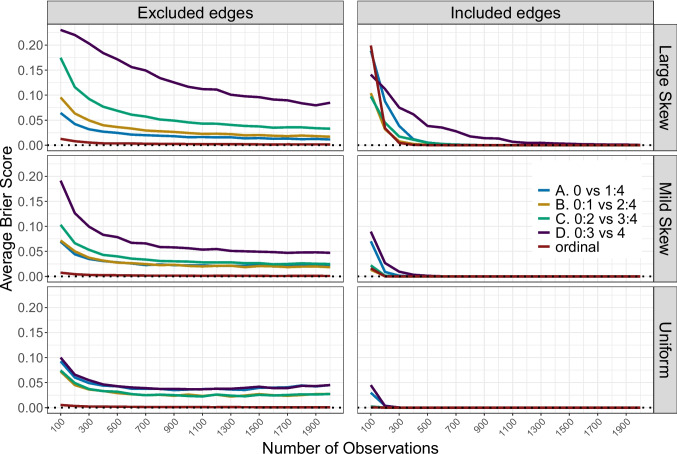
Brier scores for the accuracy of the models estimated using the ordinal data and the dichotomized data with the four different splits (indicated by different colors) are plotted as a function of the number of observations. Each row represents the different ordinal distributions condition, whereas the two columns represent the results split with respect to truly excluded and truly included edges. The *dotted black line* indicates a Brier score of zero, representing perfect recovery of the true network structure

Figure 2 depicts the same results, but now split based on whether the edges were excluded or included in the true network structure used to generate the data. The different rows correspond to the three ordinal distribution conditions. We see two important patterns. First, as before (cf., Fig. 1), the Brier score is highest for data with a large skew. Second, the inaccuracy is greater for the more extreme dichotomization splits. Notably, we now see that the inaccuracy introduced by dichotomizing the data is much more pronounced when recovering truly excluded edges (i.e., conditional independence relations) than when recovering truly included edges. For the latter, even the dichotomized data tend to reach perfect accuracy (i.e., an average Brier score of zero) with increasing sample size. This pattern is consistent across all dichotomization criteria. When analyzing the ordinal data directly, the inaccuracy quickly diminishes as the sample size increases. In contrast, the inaccuracy of models estimated on dichotomized data remains relatively stable in recovering truly excluded edges, even as the sample size increases, across all marginal distribution conditions. To explore this further, we include a condition with a sample size of 5, 000 under the Mild Skew scenario. For the truly included edges, we find that, consistent with the results for n = 2, 000, all Brier scores are zero, both for the dichotomized and ordinal data. However, the Brier scores for the truly excluded edges are still not zero. Table 1 illustrates the change in average Brier scores for the truly excluded edges as the sample size increases from 2, 000 to 5, 000. We observe that, while the Brier scores remain low for the dichotomized data, they do not reach zero and even tend to slightly increase with sample size. Therefore, even though most absent edges are eventually recovered with increasing sample size, some inaccuracy persists when using dichotomized data.

We suspect that the reduced accuracy in recovering truly excluded edges arises because, when an edge is truly absent, dichotomizing the data distorts the underlying information in a way that leads the model to erroneously detect a spurious association. This may be explained by the reduction in dimensionality of the threshold parameters (see Appendix A) when estimating the model on dichotomized data, which can influence parameter estimation and increase the likelihood of false positives. As a result, the model may incorrectly identify certain edges as included, even though they are excluded in the true underlying network structure from which the ordinal data were generated. This also explains why, in the analysis of the ordinal data, there is no such discrepancy between the Brier scores for included and excluded edges.

**Table 1:** Average Brier scores for the accuracy of recovering excluded edges under dichotomized data with the four different splits and the ordinal data

	n = 2, 000	n = 5, 000
A. 0 vs 1:4	0.0222	0.0431
B. 0:1 vs 2:4	0.0184	0.0319
C. 0:2 vs 3:4	0.0248	0.0355
D. 0:3 vs 4	0.0472	0.0570
Ordinal	0.0009	0.0000

These results pertain only to the mild skew condition for ordinal categories, under sample sizes of 2, 000 and 5, 000

### Rescaling the edge weight parameters

When modeling ordinal variables using the OMRF, applying the same prior settings to all edges can introduce bias because the interaction terms for ordinal variables span a wider range. In Appendix C, we address the question of rescaling the edge weight parameters and examine how this impacts the accuracy of results, particularly when analyzing ordinal data. We demonstrate that standardizing the parameters leads to a slight improvement in accuracy with regard to correctly identifying truly excluded edges.

## Discussion

In this paper, we have demonstrated that modeling multivariate ordinal data using network models at their original measurement levels yields more accurate recovery of the true network structure compared to dichotomizing the data and employing an Ising model. Our simulation study showed that the ordinal MRF consistently outperforms dichotomization in accurately recovering the true network structure across varying sample sizes and distributions of ordinal variables.

The results of our study can be summarized as follows. Across all simulation conditions, models estimated using the ordinal data consistently outperform those based on dichotomized data, with especially large differences in accuracy when the distributions of the ordinal categories are skewed. The ordinal MRF reliably recovers the true network structure and achieves near-perfect performance as the sample size increases, regardless of the distribution of the ordinal variables and the true inclusion of the edges. In contrast, the models estimated on dichotomized data exhibited greater inaccuracy, particularly under extreme dichotomization criteria and especially when recovering truly excluded edges. While accuracy in recovering included edges improved with larger sample sizes across all methods, identifying conditional independence relations (i.e., absent edges) remained considerably more challenging for dichotomized data, with slight inaccuracies persisting even at quite large sample sizes. These results suggest that dichotomizing the data asymmetrically distorts the evidence, disproportionately weakening the signal for excluded edges relative to that for included edges.

The results highlight the importance of selecting an appropriate modeling approach for ordinal data. While dichotomization may offer convenience, it often comes at the cost of reduced accuracy. The observed inaccuracies in network recovery using dichotomized data are driven by both the choice of cutoff and the distributional properties of the ordinal variables. When the ordinal variables are skewed, applying extreme dichotomization cutoffs can result in severely imbalanced splits, leading to variables with very low variance. This, in turn, makes both estimation and statistical testing more difficult and unreliable. These issues are particularly problematic when attempting to detect excluded edges, as dichotomization disproportionately distorts information about conditional independence relations. Although more balanced dichotomization cutoffs (and more uniform data distributions) can mitigate some of these challenges, the loss of information remains an inherent limitation of the approach. In contrast, the ordinal MRF provides a principled framework for modeling ordinal variables directly, avoiding ad hoc transformations and preserving the structure of the data. From a practical standpoint, whether the inaccuracies introduced by dichotomization are tolerable depends on the research objective. In studies focused on identifying strong included associations, dichotomization may be acceptable. However, for applications that require accurate recovery of the full network structure, including conditional independence relations, the ordinal MRF is a more robust and informative alternative. We strongly believe that the availability of the ordinal MRF (Marsman et al., 2025), user-friendly software to estimate this model (Marsman et al., 2025), and accessible tutorial papers (e.g., Huth et al., 2023; Sekulovski, Keetelaar, Haslbeck, & Marsman, 2024) alleviates the need to dichotomize ordinal data.

In conclusion, our findings suggest that researchers using network psychometric models stand to gain substantially by adopting the ordinal MRF, which offers a more accurate and principled approach to structure recovery than dichotomizing ordinal data and applying the Ising model. By leveraging the ordinal MRF, researchers can achieve more reliable and nuanced insights into the conditional (in)dependence structure of psychological variables, ultimately enhancing the validity of their network analyses.

## Figures and Tables

**Fig. 3:**
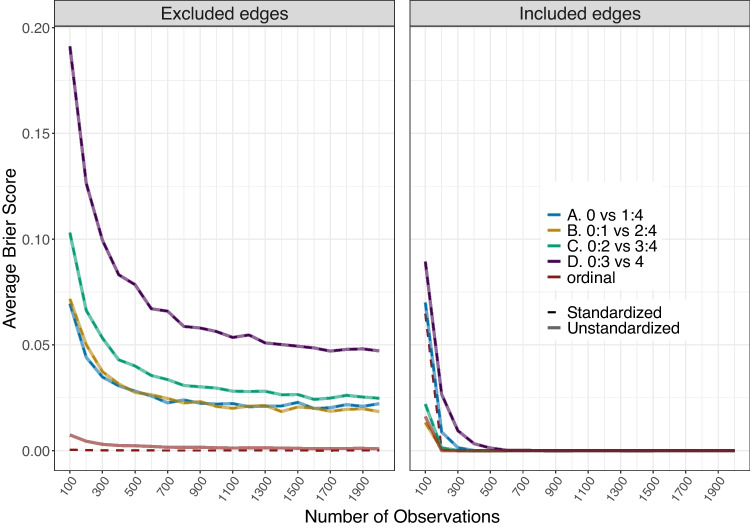
Brier scores for the accuracy of the models estimated for the ordinal data and the dichotomized data with the four different splits (indicated by *different colors*) are plotted as a function of the number of observations, for data where the ordinal categories exhibit mild skew. The *solid faded lines* are the results from the main simulation (cf., second row of Fig. 2), whereas the *dashed lines* represent the new results with standardization. The two columns represent the results split with respect to truly excluded and truly included edges

## Data Availability

Not applicable.

## Appendix A: The Bayesian analysis of the ordinal Markov random field

Suppose we have *p* variables for *n* observations, with each variable $X_i$ for $i = 1, \dots , p$ having $m_i + 1$ ordinal response categories with possible realizations $x_i = 0, \dots , m_i$. The ordinal MRF, introduced by Marsman et al. , (2025), is defined as the joint distribution of a vector $\textbf{X}$ of *p* variables

1 $$\begin{aligned} p(\textbf{X} \mid \boldsymbol{\mu }\text {, }\boldsymbol{\Theta })= &  \frac{1}{\text {Z}} \exp \left( \sum _{i=1}^p \sum _{h=1}^{m_i}\mathcal {I}(X_i = h) \mu _{ih}\right. \left. + 2\sum _{i=1}^{p-1}\sum _{j=i+1}^pX_iX_j\theta _{ij}\right) ,\nonumber \\ \end{aligned}$$

where $\mu _{ih}$ denotes the category threshold for category *h* (for $h = 0, \dots , m$) for variable *i* and $\mathcal {I}(.)$ is an indicator function that is equal to one if its condition is met and zero otherwise. The edge weight parameters $\theta _{ij}$, stored in the symmetric matrix $\boldsymbol{\Theta }$, are of primary interest to researchers as they describe the strength of the pairwise association between the included edges in the network between variables *i* and *j*. It is important to note that when $m = 1$, for all variables, the ordinal MRF is equivalent to the Ising model. Finally, Z represents the normalizing constant, which assures that the distribution sums to unity; say that all *p* variables have *m* response categories, then the space of all possible response patterns will have $m^p$ terms, which becomes computationally very expensive as *p* and *m* grow. Therefore, the authors resort to using the pseudolikelihood (Besag, 1974) as an approximation to the full likelihood. The pseudolikelihood is defined as the product of the full conditional distributions of each variable given the remaining variables and therefore reduces the terms in the normalizing constant to a maximum of $m\times p$. We refer the interested reader to the main paper by Marsman et al. , (2025) for the details on the model and to Keetelaar, Sekulovski, Borsboom, and Marsman (2024) for a discussion on whether the (joint) pseudolikelihood for the Ising model is a good approximation to the full likelihood. The ordinal MRF and its Bayesian analysis described below are implemented in the R package bgms (Marsman et al., 2023).

A network of *p* variables has $k = p(p-1)/2$ possible edges, which gives $2^k$ possible structures, meaning that as the number of variables grows, we become quite uncertain about the network structure. BMA is a robust approach to account for this uncertainty, achieved by specifying prior distributions for both the graph/network structure and the edge weight parameters. Under BMA the joint posterior distribution of the network structure and edge weight parameters is given by:

$$\begin{aligned} \underbrace{p(\boldsymbol{\mu } \text {, }\boldsymbol{\Theta }\text {, }\mathcal {S} \mid \text {data})}_{\text {joint posterior}}\propto &  \underbrace{p^*(\text {data} \mid \boldsymbol{\mu } \text {, }\boldsymbol{\Theta } \text {, }\mathcal {S})}_{\text {pseudolikelihood}} \times \underbrace{p(\boldsymbol{\mu })}_{\begin{array}{c} \text {prior on the} \\ \text {category thresholds} \end{array}} \times \underbrace{p(\boldsymbol{\Theta }\mid \mathcal {S})}_{\begin{array}{c} \text {prior on the} \\ \text {edge weights} \end{array}} \times \underbrace{p(\mathcal {S})}_{\begin{array}{c} \text {prior on the} \\ \text {graph structure} \end{array}}. \end{aligned}$$

Where $p(\mathcal {S})$ represents the prior on the network structure, which determines the prior on the edge weight parameter $p(\boldsymbol{\Theta }\mid \mathcal {S})$. The default prior setup in the R package bgms adopts a uniform prior for the graph structure; meaning that each edge is assigned an independent Bernoulli distribution with a prior inclusion probability of 0.5. Additionally, a discrete spike and slab prior is employed for the edge weight parameters, specified as a Cauchy distribution with a scale of 2.5 for the slab component. For a comprehensive introduction to the Bayesian analysis of MRF models, we refer the interested reader to Huth et al. , (2023). Further insights into the prior distributions concerning the network structure and edge weight parameters can be found in Sekulovski et al. , (2024).

One of the main advantages of using MRF graphical models is that a included edge in the network represents a conditional dependence relationship between the pair of variables after having accounted for the remaining variables in the network (Kindermann & Snell, 1980). To infer conditional independence relationships, we can compute posterior inclusion probabilities for all the edges. Using these posterior inclusion probabilities, we can quantify the evidence provided by the data for the inclusion of an edge between two variables *i* and *j* (i.e., the alternative hypothesis ($\mathcal {H}^{(ij)}_1$)) versus the evidence for its exclusion (i.e., the null hypothesis $(\mathcal {H}^{(ij)}_0$)). The posterior inclusion probability of the edge between variables *i* and *j* can be expressed as the sum over the posterior probabilities of all structures $\mathcal {S}^\prime$ that contain an edge between variables *i* and *j*, i.e., for which $\mathcal {H}^{(ij)}_1$ is true

$$ p(\mathcal {H}^{(ij)}_1 \mid \text {data}) = \sum _{\mathcal {S}^\prime \in \mathcal {S}^{(i-j)}}p(\mathcal {S}^\prime \mid \text {data}). $$

For example, one can include all edges with a posterior inclusion probability $\ge 0.5$ (known as the median probability model, Barbieri & Berger, 2004), or use a different threshold. Another approach is to formally test for conditional independence using the inclusion Bayes factor, which can quantify the relative evidence for $\mathcal {H}^{(ij)}_1$ vs $\mathcal {H}^{(ij)}_0$. The inclusion Bayes factor is defined as

$$\begin{aligned} \text {BF}_{10} = \underbrace{\frac{p(\mathcal {H}^{(ij)}_1\mid \text {data})}{p(\mathcal {H}^{(ij)}_0 \mid \text {data})}}_{\begin{array}{c} \text {Posterior}\\ \text {inclusion odds} \end{array}} \bigg / \underbrace{\frac{p(\mathcal {H}^{(ij)}_1)}{p(\mathcal {H}^{(ij)}_0)}}_{\begin{array}{c} \text {Prior}\\ \text {inclusion odds} \end{array}}. \end{aligned}$$

For the results in this paper, we only use the posterior inclusion probability; we refer the interested reader to Sekulovski et al. , (2024) for a detailed discussion on Bayesian tests for conditional independence testing in network psychometrics.

## Appendix B: Simulating ordinal data

We generate the data from the ordinal MRF with three different distributions for the ordinal categories by varying only the category thresholds $\mu _{i,h}$ and holding the edge-weight matrix, $\boldsymbol{\Theta }$, fixed. In particular, we define the marginal probabilities on the ordinal categories $\{0,1,\dots ,m\}$ by

$$\begin{aligned} p_h \;=\; \frac{e^{-\alpha \,h}}{\displaystyle \sum _{u=0}^{m} e^{-\alpha \,u}}, \qquad h = 0,1,\dots ,m, \end{aligned}$$

and then set the thresholds for each node *i* and level $h=1,\dots ,m$ as

$$\begin{aligned} \mu _{i,h} = \log \!\bigl (p_h/p_0\bigr ) \;-\; \frac{m}{2}\,h\;\sum _{j\ne i}\theta _{ij}. \end{aligned}$$

Therefore, when $\alpha =0$, all $p_h = 1/(m+1)$, yielding a uniform distribution. As $\alpha$ grows, $p_0>p_1>\cdots >p_m$, giving progressively stronger right-skew.

For the edge weight parameter matrix $\boldsymbol{\Theta }$, we generate an adjacency matrix with about $10\%$ of the edges included; all included edges were given a partial association parameter of 0.25.

## Appendix C: Adjusting the scale of the prior distribution on the edge weight parameters

Since the parameters of the ordinal MRF are not standardized by default, applying the same scale hyperparameter to the prior distribution of all edge weight parameters can introduce bias. As shown in Eq. 1, the product $X_i \times X_j$ can range from zero up to $m_i \times m_j$ for ordinal variables, while for binary variables, it can only be zero or one. If the prior distribution for the edge weights (i.e., the Cauchy slab) is given the same scale for both ordinal and binary edges, it fails to account for the larger possible range of the ordinal interaction terms. To correct for this, we implement a standardization in which the prior scale for each interaction is multiplied by the product of the number of categories of the two variables. This ensures that the prior is appropriately scaled for each edge, making the edge weight estimates comparable across variable types. This behavior is related to the Jeffreys–Lindley paradox (Lindley, 1957); a mismatch between the scale of the prior and the number of ordinal categories of the variables can result in larger evidence for the null hypothesis (i.e., the hypothesis in favor of conditional independence). For more details, see Marsman et al. , (2025) and Sekulovski et al. , (2024). We have implemented this standardization procedure in the R package bgms.

We re-ran the simulation for the condition with a mild skew, the results are presented in Fig. 3. For binary data, the performance of standardized and unstandardized models is virtually identical, regardless of whether edges are included or excluded. In contrast, for ordinal data, standardization offers a slight advantage for excluded edges, achieving lower Brier scores and thus more accurate identification of excluded edges across all sample sizes. This is evident from the difference between the solid and dashed red lines. However, for included edges at low sample sizes, the standardized approach underperforms relative to the unstandardized model, showing higher Brier scores. This is because standardization increases the prior scale for interactions between ordinal variables; the prior becomes broader and less informative. With little data, this leads us to favor edge exclusion (i.e., the null hypothesis of conditional independence). In contrast, the unstandardized (narrower) prior can yield higher inclusion probabilities. As the sample size increases, the influence of the prior diminishes.

## Footnotes

1 Many of these methods are also implemented in the statistical software JASP (JASP Team, 2025).

2 Note that these distributions are subject to random variation and will not be exactly uniform—that is, not every ordinal category will have an equal number of observations. However, their approximate uniformity makes the distributions more realistic. See also Fig. 1.

## Code Availability

The data and materials for all simulation examples are available at the OSF repository https://osf.io/9bzet/.
